# Understanding community as a basis for targeting and shaping service delivery

**DOI:** 10.1002/jia2.25712

**Published:** 2021-06-30

**Authors:** Keletso Makofane, Richard Lusimbo, Pascale Macharia, Olumide Makanjuola, Shawn Malone, Augustus Mokabedi, Steave Nemande, Cheikh Traore, Jeffrey Walimbwa

**Affiliations:** ^1^ FXB Center for Health and Human Rights Harvard University Boston MA USA; ^2^ Global Black Gay Men Connect New York NY USA; ^3^ Pan Africa ILGA Kampala Uganda; ^4^ HOYMAS Nairobi Kenya; ^5^ ISDAO Lagos Nigeria; ^6^ PSI Johannesburg South Africa; ^7^ LEGABIBO Gaborone Botswana; ^8^ Evolve Yaoundé Cameroon; ^9^ Independent Consultant Lagos Nigeria; ^10^ ISHTAR MSM Nairobi Kenya

**Keywords:** MSM, community, LGBT, services

Despite the expansion of HIV prevention and treatment services across sub‐Saharan Africa, new HIV infections remain stubbornly high [1]. It has been suggested that this is a result of the failure to extend services to groups such as men who have sex with men, who have a high risk of acquiring and transmitting HIV [2]. In this Viewpoint, we argue that community‐based organizations, which are largely responsible for delivering services to men who have sex with men, should be understood in relation to the communities from which they emerge and in which they are embedded. This understanding can help HIV programmes to improve the reach of their services and expose gaps in services.

In global guidance, “community” is an important organizing principle for the HIV response. Communities are thought to be vehicles for designing and delivering appropriate, contextualized services and mitigating or modifying structural barriers to health and barriers to access to healthcare [3, 4]. The idea of community is inherently social. It signifies a group that is bound by relationships among people, including relationships based on shared identity. However, the category “men who have sex with men”, or MSM, based entirely on shared behavior, does not relay any information about the relationships that might give rise to the cohesiveness of the group [5]. The phrases “community consultation,” “community‐led” and “community support,” therefore, are ambiguous in the context of men who have sex with men; the referent group remains unclear.

Guided by Aggleton and Parker [6], we define community as a group of people joined together by shared location, solidarity or shared identity. By location, we mean that community can be understood as a group of people who share space; living or working or playing in the same area or traversing the same set of physical locations. By shared identity, we mean the classification of oneself into some group and the tendency to have common experiences and shared meanings with that group. We take solidarity to mean “shared sentiment, and… the desire to work together for the better good” [6] – a bond that predicates the exchange of help or coordination of joint action. In addition, we consider social networks – connections through friendship, acquaintanceship, kinship, etc. – as a glue that binds communities together.

We argue that being clear about the role of shared identity, solidarity, locality and social networks in forming and running community‐based organizations can help to identify groups that are systematically excluded from community‐delivered services. To this end, we reflect on the professional experiences of the authors in founding, running and supporting community‐based HIV programmes for men who have sex with men across sub‐Saharan Africa.

In December of 2005, following a series of high‐profile arrests made under anti‐sodomy law, a Cameroonian newspaper published a list of “The Top 50 Presumed Homosexuals in Cameroon,” inciting explosive public discussion about same‐sex sexuality [7]. Steave Nemande, then a physician working at a private clinic, started meeting with friends to devise a response. These included sociologist Charles Gueboguo, who Nemande had met through his partner at a cocktail reception in Douala. Gueboguo had, in turn, introduced Nemande to his friend Joel Nana – an activist lawyer who lived in Nigeria but often had business in Cameroon. In 2005 these friends formed Alternatives‐Cameroun [8] to change how same‐sex sexuality is spoken about. This organization, like many others, was established as an act of solidarity by a group of friends who identified as gay.

Beyond inception, friendships play an important role in the growth of programmes and organizations. Friendship networks are an important source of clients and workers. These networks are shaped by encounters in spaces that gay men socialize in. The outreach programme at Ishtar MSM [9] in Kenya, for instance, consistently finds new clients through the friends and acquaintances of volunteer peer educators, sometimes engaging those clients as peer educators themselves and tapping into their networks. To the extent that solidarity motivates the volunteerism of peer educators, it is crucial, too, in the growth of the programme.

Shared identity is crucial to establishing the trusting relationships that community‐based organizations enjoy with their clients and members. In Uganda, Sexual Minorities Uganda [10] organizes outreach using “cells”. Each cell is a group of ten to 20 clients who are reached with information, prevention commodities and referrals by a single peer educator. Through this system, clients are linked to local clinics whose healthcare workers have been trained to provide culturally competent care. The programme matches the identity of the peer educator with those of the clients, facilitating disclosure of the client’s needs and shoring up the legitimacy of the programme in the eyes of its intended beneficiaries.

Finally, in addition to channelling resources from organizations to individuals, peer educators continually collect information about the needs of clients. In Kenya, Health Options for Young Men on HIV/AIDS/STI (HOYMAS) [11] has a target of reaching at least 80% of their clients every quarter and gathering feedback on programmes.

In summary, community‐based organizations emerge from, and are deeply embedded in, communities of people who share space, identities, solidarities and social networks.

But among the people organizations seek to serve, some are not members of these communities. These include men who have sex with men who do not identify as gay or bisexual or those who do identify, but for various reasons do not live public gay lives. For these men, the place‐based intervention strategies that are the basis of many outreach programmes likely fail; the men avoid places where they could be identified as gay. Extant network‐based outreach programmes are also unlikely to be successful; men who do not want to be identified as gay intentionally limit their social connections with those who do. For the same reason, these men may not volunteer to be peer educators as frequently as gay‐identified men. They may not feel solidarity.

Beyond identity, a multitude of factors shapes inclusion or exclusion in communities. Men who live in rural areas, for instance, might identify as gay but not be connected to the social networks, solidarities and physical spaces that urban gay men are. Counterintuitively, though affluent men are more likely to afford healthcare, they may have diminished access to information and services tailored to men who have sex with men since these are often targeted at men who are not affluent.

Men who do not regularly make contact with community‐based organizations or frequent the places where they conduct activities are not necessarily sealed off from those organizations. For example, in Nairobi there are groups of men who socialize in private social spaces with purposefully small, closed social networks. Community‐based organizations are not invited to these spaces most of the time, but individuals often know how to reach organizations in times of need – for post‐exposure prophylaxis or assistance in dealing with a blackmailer.

Beyond this ad hoc contact, a number of organizations have attempted interventions to regularly reach men who are outside communities. These range from holding support groups separately for men who do not identify as gay to targeting services to men who live in more affluent areas. These programmes have generally not been implemented at scale.

Social networks, shared identity, locality and solidarity are crucial to the functioning of community‐based organizations. By examining these aspects of community, organizations can predict which groups of individuals are left out of their programmes. There is a need for further research on the functioning of community‐based organizations and heterogeneity in the ways individuals interact with these institutions.

The category of men who have sex with men needs to be disaggregated not only for the purpose of understanding the epidemiology of HIV [12], but also for understanding access to and usage of services. There might never be an organization led by non‐gay‐identifying men who have sex with men. Organizations in the HIV response, however, can cultivate networks of non‐gay‐identifying men and extend services through them. The challenge of programming for the future lies not only in accounting for the multitude of identities of members, but also in understanding and shaping the identity of services.

## Competing interests

The authors have no conflicts of interest to declare.

## Authors’ contributions

KM, RL, PM, OM, SM, AM, SN, CT and JW participated in discussions whose content formed the basis of this manuscript. KM drafted the manuscript.
